# Differential impact of eicosapentaenoic acid and docosahexaenoic acid in an animal model of Alzheimer’s disease

**DOI:** 10.1016/j.jlr.2024.100682

**Published:** 2024-10-28

**Authors:** Méryl-Farelle Oye Mintsa Mi-mba, Meryem Lebbadi, Waël Alata, Carl Julien, Vincent Emond, Cyntia Tremblay, Samuel Fortin, Colin J. Barrow, Jean-François Bilodeau, Frédéric Calon

**Affiliations:** 1 Faculty of Pharmacy, Laval University, Quebec, QC, Canada; 2 Centre Hospitalier de l'Université Laval (CHUL) Research Center, Quebec, QC, Canada; 3 Centre de recherche sur les biotechnologies marines, Rimouski, QC, Canada; 4 Centre for Sustainable Bioproducts, Deakin University Geelong, Victoria, Australia; 5 Department of medicine, Faculty of Medecine, Laval University, Quebec, QC, Canada

**Keywords:** 3xTg-AD mice, Alzheimer’s disease, eicosapentaenoic acid, docosahexaenoic acid, *n*-3 polyunsaturated fatty acids

## Abstract

Dietary supplementation with n-3 polyunsaturated fatty acids improves cognitive performance in several animal models of Alzheimer's disease (AD), an effect often associated with reduced amyloid-beta and/or tau pathologies. However, it remains unclear to what extent eicosapentaenoic (EPA) provides additional benefits compared to docosahexaenoic acid (DHA). Here, male and female 3xTg-AD mice were fed for 3 months (13–16 months of age) the following diets: (1) control (no DHA/EPA), (2) DHA (1.1g/kg) and low EPA (0.4g/kg), or (3) DHA (0.9g/kg) with high EPA (9.2g/kg). The DHA and DHA + EPA diets respectively increased DHA by 19% and 8% in the frontal cortex of 3xTg-AD mice, compared to controls. Levels of EPA, which were below the detection limit after the control diet, reached 0.14% and 0.29% of total brain fatty acids after the DHA and DHA + EPA diet, respectively. DHA and DHA + EPA diets lowered brain arachidonic acid levels and the n-6:n-3 docosapentaenoic acid ratio. Brain uptake of free ^14^C-DHA measured through intracarotid brain perfusion, but not of ^14^C-EPA, was lower in 3xTg-AD than in NonTg mice. DHA and DHA + EPA diets in 3xTg-AD mice reduced cortical soluble phosphorylated tau (pS202) (−34% high-DHA, −34% DHA + EPA, *P* < 0.05) while increasing p21-activated kinase (+58% and +83%, *P* < 0.001; respectively). High EPA intake lowered insoluble phosphorylated tau (−31% vs. DHA, *P* < 0.05). No diet effect on amyloid-beta levels was observed. In conclusion, dietary intake of DHA and EPA leads to differential changes in brain PUFA while altering cerebral biomarkers consistent with beneficial effects against AD-like neuropathology.

Alzheimer's disease (AD) is a neurodegenerative disorder that causes progressive and irreversible loss of mental functions (1, 2). Since AD is the main cause of dementia in the elderly and its prevalence is rising rapidly with the aging of the population, using nutraceutical compounds such as n-3 long-chain polyunsaturated fatty acids (PUFA) to lower AD prevalence is attracting a lot of interest (3, 4, 5). PUFA are mainly derived from the diet and are important in maintaining human health. The shift to a higher ratio of omega-6 to omega-3 PUFA (n-6: n-3 PUFA ratio) in Western diets over the past centuries is a topic of concern, given the well-known role of n-3 PUFA in the maintenance of learning ability and memory in animals (6, 7, 8, 9). Preclinical studies have shown that both free docosahexaenoic acid (DHA) and eicosapentaenoic acid (EPA) cross the blood-brain barrier (BBB) through nonsaturable uptake mechanisms (10, 11, 12) and that a diet rich in n-3 PUFA reduces amyloid-beta (Aβ) (13, 14, 15, 16) and tau pathologies (16, 17), increases postsynaptic markers (15, 18, 19, 20), and improves cognitive performance (6, 14, 18) in several animal models of AD. Most epidemiological studies suggest that a low intake or low plasma levels of n-3 PUFA are associated with an increased incidence of AD (21, 22, 23, 24, 25).

While results from randomized controlled trials lend limited support for the use of n-3 PUFA in AD prevention, many confounding variables have been identified and the exact formulation that is most likely to benefit patients remains yet to be determined (4, 5, 26, 27, 28). PUFA concentrations in the brain are characterized by high levels of arachidonic acid (ARA) and DHA, but very low EPA content (19, 29, 30). With its ability to readily incorporate into cell membranes of neurons, dietary DHA (n-3 PUFA) can modulate key aspects of brain function, including membrane fluidity, cell signaling, and gene expression (18, 31, 32). Also found in fish oil, EPA is another n-3 PUFA that has been postulated to play a role in brain functions particularly via a reduction of pro-inflammatory ARA (n-6 PUFA) derivatives (9, 33). EPA is a potent anti-inflammatory agent and can displace ARA in immune cell membranes to affect T-cell signaling and antigen presentation (34, 35).

Since the brain is unable to synthesize DHA and EPA de novo, it is necessary that it obtains these essential fatty acids through diet or from their precursor α-linolenic acid (36). However, it remains uncertain whether the neuroprotective effects of EPA are comparable to those of DHA following oral administration.

Despite its low concentration and extensive β-oxidation upon entry into the brain, EPA has significant and important biological activity which differs from that of DHA (12, 37, 38, 39, 40, 41). Clinical trials have shown effects of EPA on gene expression (42), vascular function (43), mood disorders such as depression (44, 45, 46), sleep deficiency (47), and cognitive functions (4, 48, 49, 50). Some clinical trials (51, 52), but not all of them (53, 54, 55), have reported different effects of DHA and EPA supplementation on inflammation markers. Recent clinical and preclinical evidence have suggested that EPA may have additional effects compared to DHA, notably on cardiovascular diseases (56, 57, 58, 59), estimation of brain atrophy (60), and mood disorders, although not without contradictory findings (46, 57). It has been proposed that differential effects of EPA versus DHA, notably in the brain, comes from downstream derivatives (6, 12, 45, 61). From a molecular standpoint, EPA is the precursor of classes of anti-inflammatory and anti-thrombotic eicosanoids (prostaglandins, isoprostane, prostacyclin, and thromboxane), whereas DHA is a precursor of neuroprotectins, D-series resolvins, and maresins (62, 63, 64, 65). Nonetheless, whether dietary EPA and DHA exert differential effects in neurodegenerative diseases remains a relatively open question.

To understand the difference between the specific mechanisms of action of DHA and EPA in the brain, 3xTg-AD mice modeling AD neuropathology and age-matched NonTg mice were fed with three different diets from the age 13–16 months: a control diet without DHA nor EPA, a DHA-enriched diet (∼0.1 g .kg^−1^.day^−1^) with low EPA (∼0.05 g kg^−1^.day^−1^), or a DHA-enriched diet (∼0.08 g .kg^−1^.day^−1^) with high EPA (∼1 g kg^−1^.day^−1^). Endpoints included intact fatty acid profiles, oxidized fatty acid (isoprostanes) profiles, prostaglandins, as well as the extent of Aβ, tau, and synaptic pathologies in the brain cortex of 3xTg-AD mice. In addition, the uptake of EPA and DHA across the BBB was directly investigated using in situ brain perfusion.

## Materials and methods

### Animals and diets

Table 1 provides a detailed description of the diets and their fatty acids content determined as explained below. The microencapsulated powders enriched in DHA or in EPA were a generous gift from Ocean Nutrition Canada (now part of DSM) and were incorporated in rodent pelleted diets prepared by Research Diets (New Brunswick, New Jersey). All three diets were formulated to contain equivalent contents of linoleic acid (1) and cholesterol. However, as a new method to produce microencapsulated PUFA from fish oil was used, the proportions of ARA, DHA, and EPA could not be predetermined perfectly. The exact levels of fatty acids in the pelleted diets were thus determined by gas chromatography once the diets were prepared.

**Table 1:** Comparison of dietary treatments

Diets	Control		DHA		DHA + EPA	
	% (w/w)	% (cal/cal)	% (w/w)	% (cal/cal)	% (w/w)	% (cal/cal)

Proteins	24.0	23	23.7	19	23.7	19
Carbohydrates	45.2	34	45.4	34	45.4	34
Fats	20.4	43	20.5	46	20.5	46
kcal/g		4.25		4.23		4.23

**Ingredients**	**g**	**kcal**	**g**	**kcal**	**g**	**kcal**

Casein	200	800	170	680	170	680
DL-methionine	3	12	3	12	3	12
Corn Starch	75	300	75	300	75	300
Sucrose	223	892	223	892	223	892
Cellulose, BW200	50	0	50	0	50	0
Safflower oil	40	360	35	315	35	315
Butter, anhydrous	92	828	72	648	72	648
Lard	41	369	33	297	33	297
Microencapsulated DHA or EPA	0	0	60	390	60	390
Cholesterol, USP	1.5	0	1.3	0	1.3	0
Ethoxyquin	0.001	0	0.001	0	0.001	0
Minerals (S19101)	0	0	0	0	0	0
Vitamins (V15908)	10	40	10	40	10	40
Choline Bitartrate	2	0	2	0	2	0
**Total**	847.6	**3601**	844.4	**3574**	844.4	**3574**

**Fatty acids (FA) content** (as determined by gas chromatography in the pelleted diet)			
	g/kg	g/kg	g/kg
C18:2 n-6 LA	29.0	29.9	29.1
C20:4 n-6 ARA	0.2	0.5	0.8
C22:4 n-6 DTA	0.0	0.0	0.0
C22:5 n-6 DPA	0.0	0.0	0.0
**Total n-6 PUFA**	29.2	30.3	30.0
C18:3 n-3 ALA	0.6	0.6	0.7
C20:5 n-3 EPA	**0**	**0.4**	**9.2**
C22:5 n-3 DPA	**0**	**0.5**	**1.8**
C22:6 n-3 DHA	**0**	**1.1**	**0.9**
**Total n-3 PUFA**	**0.6**	**2.1**	**10.7**

ALA, alpha linolenic acid; ARA, arachidonic acid; DHA, docosahexaenoic acid; DPA, docosapentaenoic acid; DTA, docosatetraenoic acid; EPA, eicosapentaenoic acid; FA, fatty acids; LA, linoleic acid; PUFA, polyunsaturated fatty acids.

The use of animals was approved by the Laval University Animal Research Ethics Committee in accordance with the standards of the Canadian Council on Animal Care. Triple-transgenic mice (3xTg-AD, B6;129-Tg(APPSwe,tauP301L)1Lfa *Psen1^tm1Mpm^*) harboring three mutant genes, namely APP_swe_, PS1_M146V_, and tau MAPT_P301L_, were from a colony maintained in our animal facilities and generated from founder mice obtained from Dr Frank LaFerla (66). Transgenic mice were compared with nontransgenic (NonTg) littermates with the same genetic background (C57BL6/129SvJ). Three groups of 3xTg-AD mice were fed with one of the three different diets for 3 months: (1) a control diet (without DHA nor EPA) (seven males and five females), (2) a DHA-enriched diet (1.1 g or 3.4 mmol per kg of diet, corresponding to a dose of ∼0.1 g .kg^−1^.day^−1^) with low EPA (0.4 g or 1.3 mmol per kg of diet, corresponding to a dose of ∼0.05 g .kg^−1^.day^−1^) (seven males and seven females), or (3) a DHA-enriched diet (0.9 g or 2.7 mmol per kg of diet, corresponding to a dose of ∼0.08 g .kg^−1^.day^−1^) with high EPA (9.2 g or 30.4 mmol per kg of diet, corresponding to a dose of ∼1 g .kg^−1^.day^−1^) (seven males and six females). Beside DHA and EPA, it is also worth mentioning that ARA (+60%) and n-3 docosapentaenoic acid (DPA) (3.6-fold) were also present in greater amounts in the EPA + DHA diet than in the DHA-enriched diet (Table 1). Because of the limited availability of the EPA formulation, NonTg mice were fed (1) the control diet (six males and four females) or (2) the DHA diet with low EPA (five males and five females) for 3 months, but not the EPA-rich diet. Mice received diets starting at the age of 13 months and were sacrificed at 16 months of age under deep anesthesia with ketamine/xylazine and perfused via transcardiac infusion with PBS (1 mM KH_2_PO_4_, 10 mM Na_2_HPO_4_, 137 mM NaCl, 2.7 mM KCl, pH 7.4) containing a cocktail of protease inhibitors (SIGMAFAST™ Protease Inhibitor Tablets, Sigma-Aldrich, St. Louis, MO) along with phosphatase inhibitors (50 mM sodium fluoride and 1 mM sodium pyrophosphate). Frozen extracts of the frontal cortex (for fatty acid profiles), hippocampus (for isoprostane measurements), and the parieto-temporal cortex (for protein fractionation) were dissected and kept at −80°C.

### Fatty acid determination in diets

**Fig. 1:**
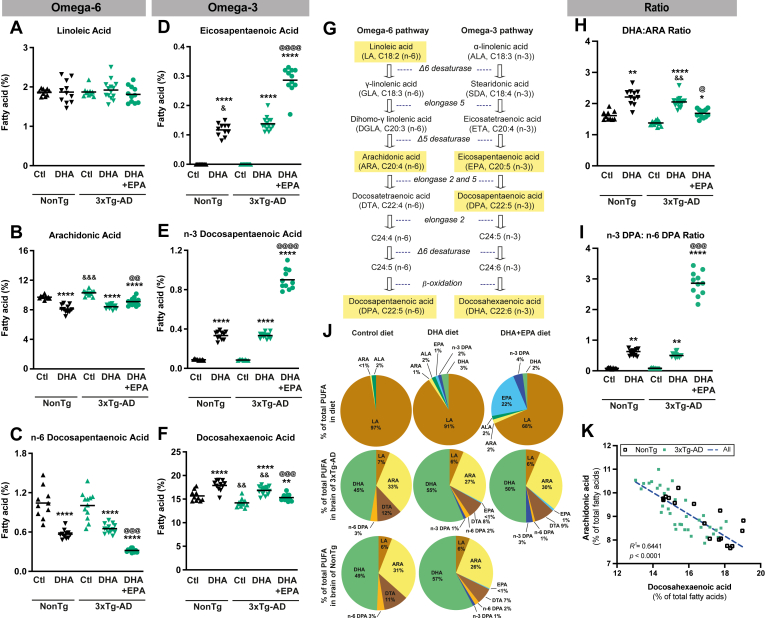
DHA and DHA + EPA-enriched diets greatly, but differentially, impact brain fatty acid profiles. (A–F) fatty acid profiles of n-3 and n-6 PUFA in the frontal cortex of both nontransgenic and 3xTg-AD mice. (A) linoleic acid levels remained unchanged following DHA and DHA + EPA supplementations. (B) both DHA and DHA + EPA diets decreased ARA levels compared to control diet. DHA + EPA diet decreased (C) n-6 DPA levels but increased both (D) EPA and (E) n-3 DPA levels in the frontal cortex of transgenic mice to a greater extent that the DHA diet, compared to control diet. (F) both DHA and DHA + EPA diets increased significantly DHA levels compared to control diet. (G) schematic representation of n-6 and n-3 PUFA pathways. Compared to control diets, (H) ratios of DHA:ARA were higher following DHA and DHA + EPA diets, but DHA + EPA diet led to less important ratio than DHA diet in transgenic mice. (I) DHA + EPA significantly increased n3 DPA:n-6 DPA ratio compared to both control and DHA diets, while DHA diet slightly increased the ratio compared to control diet in transgenic mice. (J) comparison of percentage of each PUFA in the diets versus the brain of NonTg and 3xTg-AD mice. (K) correlations between cerebral levels of ARA and DHA. Data are presented as means ± SEM, each dot represents individual values (n = 8–14 mice per group). Statistical analyses were performed using an ANOVA followed by Tukey-Kramer post-hoc tests ∗*P* < 0.05; ∗∗*P* < 0.01; ∗∗∗∗*P* < 0.0001 compared to animals with the same genotype but fed control diet. ^@^*P* < 0.05; ^@@^*P* < 0.01; ^@@@^*P* < 0.001; ^@@@@^*P* < 0.0001 compared to animals with the same genotype but fed DHA diet, ^&^*P* < 0.05; ^&&^*P* < 0.01; ^&&&^*P* < 0.001 compared to nontransgenic mice on the same diet. ARA, arachidonic acid; DHA, docosahexaenoic acid; DPA, docosapentaenoic acid; DTA, docosatrienoic acid; EPA, eicosapentaenoic acid; NonTg, nontransgenic mice; PUFA, polyunsaturated fatty acid; SEM, standard error mean; 3xTg-AD, transgenic mice.

Fatty acid profiles of diet pellets were determined using gas chromatography with flame ionization detection after being extracted with CHCl_3_:MeOH, as described (16). Then, approximately 20 mg of diet pellets were homogenized with butylated hydroxytoluene-MeOH (Sigma, St. Louis, MO) and with 22:3 n-3 methyl ester as an internal standard (Nu Chek Prep, Elysian, MN) at a concentration of 500 μg/g of diet. One and a half milliliters of NaCl 0.9% was added for homogenization, and this procedure was repeated twice. Six volumes of CHCl_3_ (J.T. Baker, Phillipsburg, NJ) and four volumes of MeOH-butylated hydroxytoluene (J.T. Baker, Phillipsburg, NJ) were added to the resulting homogenate. After centrifugation at 1500 rpm for 15 min at 4°C, the bottom layer was collected (67) and dried with a stream of nitrogen. Lipid extracts were transmethylated with benzene-MeOH (Alltech, State College, PA) and 200 μl of acetyl chloride at 100°C for 1.5 h. After cooling, 5 ml of 6% (w/v) K_2_CO_3_ were added. After centrifugation at 1500 rpm for 15 min at room temperature, the upper layer was collected (68). Benzene was dried down to about 100 μl, transferred to a gas chromatography autosampler vial, and capped under nitrogen. Fatty acid methyl ester profiles were obtained by capillary gas chromatography using a temperature gradient on a HP5890 gas chromatograph (Hewlett Packard, Toronto, Canada) equipped with a HP-88 capillary column (100 m × 0.25 mm i.d. x 0.20 μm film thickness; Agilent Technologies) coupled with a flame ionization detector. Helium was used as a carrier gas (split ratio 1:80). Fatty acids were identified according to their retention time, using the following standard mixtures as a basis for comparison: the FAME 37 mix (Supelco Inc., Bellefonte, PA) and the GLC-411 fatty acid mix (NuChek Prep Inc, Elysian, MN), as well as the following methylated fatty acids C22:5n*-*6 (Larodan AB, Malmö, Sweden) and C22:5n*-*3 (Supelco Inc., Bellefonte, PA). Finally, a mixture of trans fatty acids containing: C18:2n*-*6 cis/trans (Supelco Inc Bellefonte, PA), a mixture of cis/trans C18:3n*-*3 (Supelco Inc Bellefonte, PA), the following Nucheck fatty acids: C14:1 trans-9, C16:1 trans-9, and finally isoforms of C18:1 (cis-6, trans-6, cis-11, trans-11: Nucheck, and cis-12, cis-13, Supelco Inc.) were also used as a standard mixture. Fatty acids were expressed as g per kg of wet matter (Table 1) or as percentage of total fatty acid content (Fig. 1).

### Fatty acid determination in the brain

Fatty acids were assayed in the frontal cortex using a method coupling high-performance liquid chromatography (HPLC) to mass spectrometry (MS) (69). Fatty acids were converted to methyl esters to enable the determination of their concentrations. Briefly, 5 μl of KOH solution (9 N) were added to a 25-μl aliquot (1 mg) of homogenized brain and heated at 65°C for 45 min. After cooling the samples to room temperature, 975 μl of extraction solution [150 mM ammonium formate buffer pH = 3.0/acetonitrile (25:75 v/v)] were added and the samples were vortexed for 15 s and centrifuged (12,000×*g*) for 5 min. The resulting supernatant was transferred to a HPLC vial and 5 μl were injected. Analyses were performed using an Agilent 1200 series HPLC system at room temperature with a reversed-phase column (Zorbax, C8, 50 × 2.1 mm, 3.5 μm, Agilent). The binary gradient mobile phase [mobile phase A (5 mM ammonium acetate) and mobile phase B (acetonitrile with 0.01% formic acid)] were pumped at a flow rate of 1.0 ml/min using the following gradient: 0 min (65% B), 1.5 min (65% B), 1.7 min (99% B), 2.9 min (99% B), 2.95 min (65% B), and 3.5 min (65% B). Run time was 3.5 min with a post run time of 0.5 min. The mass spectrometry detector was operated in ESI negative mode with the capillary voltage set at 3 kV, the fragmentor at 140 V, the gas temperature at 350°C, the gas flow at 13 L/min, and the nebulizer pressure at 60 psi. Data were acquired in SIM mode corresponding to the following fatty acids: C14:0, C14:1, C16:0, C16:1, C18:0, C18:1, C18:2 (LA), C18:3, C18:4, C20:0, C20:1, C20:2, C20:3, C20:4 (ARA), C20:5 (EPA), C22:0, C22:1, C22:2, C22:3, C22:4, C22:5 (DPA), C22:6 (DHA), C24:0, C24:1, C24:2, C24:3, C24:4, C24:5, C24:6. The linear calibration curves were obtained in the concentration range of 2–500 μg/ml. The Chemstation LC/MSD software Rev.B.02.01 (Agilent) was used for instrument control and data analysis (70). Fatty acids were expressed as a percentage of total fatty acids (Fig. 1A–F). Although we did not separate fatty acids bound to phospholipids from those in free form, it is known that the vast majority of fatty acids found in cerebral tissues (∼98%) are esterified into phospholipids (71).

### In situ brain perfusion technique

In situ brain perfusion involves the direct infusion of a labeled compound into the carotid artery that has been appropriately ligatured to ensure that 100% of the perfused dose runs through the cerebral vasculature, which allows quantification of the transport of labeled molecules such as fatty acids through the BBB (10). Moreover, this technique displays several advantages, such as high sensitivity, complete control over the perfusate content, and flow rate while avoiding peripheral metabolism and distribution. The surgery was performed as previously described (10). Briefly, mice were anesthetized by intraperitoneal injection of ketamine/xylazine (140/8 mg kg^−1^). Next, the right common carotid artery was catheterized with a polyethylene catheter filled with heparin (25 IU/ml) following ligation of the external branch. Before perfusion, the thorax of the animal was opened and the heart was cut, and perfusion was immediately started with a flow rate of 1.68 ml min^−1^ for 40 s after which the mouse was sacrificed by decapitation and the right cerebral hemisphere was collected. The perfusion fluid consisted of bicarbonate-buffered physiological saline (mM solute: 128 NaCl, 24 NaHCO_3_, 4.2 KCl, 2.4 NaH_2_PO_4_, 1.5 CaCl_2_, 0.9 MgCl_2_, 9 D-glucose). The solution was gassed with 95% O2/5% CO2 to obtain a pH of 7.4 and heated to 37°C. Radiolabeled tracers, [^14^C]-DHA and [^14^C]-EPA (0.3 μCi/ml, 5 μM), were used to assess DHA and EPA transfer across the BBB, respectively. The solution used to perfuse mice with [^14^C]-DHA or [^14^C]-EPA also contained [^3^H]-inulin (0.3 μCi/ml) to monitor the physical integrity of the BBB during the procedure and to evaluate the vascular volume, since inulin is a vascular space marker that does not cross the BBB. After the perfusion, the perfused hemisphere was digested with 1 ml of SOLVABLE at 50°C overnight and then 9 ml of Ultima Gold scintillation cocktail was added to the mix. A Wallac scintillation counter (Perkin- Elmer Life Sciences) was used to quantify [^14^C] and [^3^H] in the digested brain hemisphere and perfusion fluid. The brain transport coefficient (initial brain uptake clearance, Clup, μL.g^−1^.s^−1^) of [^14^C]-DHA or [^14^C]-EPA was calculated as described previously (10) from the measured volume of distribution of [^14^C]-DHA or [^14^C]-EPA, corrected with the vascular space determined with [^3^H]-inulin. The following equation was used:

Clup=Xtissue/(Cperf×T)

Where Xtissue (dpm/g) is the measured amount of [^14^C] test compound in the right hemisphere, Cperf (dpm/μl) is the test compound concentration in the perfusion fluid, and T (s) is the perfusion time. Tissue total radioactivity was corrected for ‟vascular” contamination with:

Xtissue=Xtot−(Vvasc×Cperf)

where Xtot (dpm/g) represents the total amount of labeled compound within the tissue parenchyma and the ‟vascular” compartment, Vvasc (μl.g^−1^) is the vascular volume evaluated by measuring the distribution volume of [^3^H]-inulin using the following equation: ***Vvasc = (X***_***[3H]-inulin***_/***C***_***[3H]-inulin***_***)***, in which X (dpm/g) is the total quantity of radioactivity [^3^H] found in the right cerebral hemisphere, and C (dpm/μl) is the vascular space marker concentration in the perfusate.

### Isoprostanes and prostaglandin measurements (HPLC-MS/MS)

Total (free + esterified) F_2_-isoprostanes (F_2_-IsoPs) from oxidized ARA were extracted from 16 to 20 mg of hippocampus using a modified version of the method described by Taylor *et al*. (72). The hippocampus was homogenized with 20 μl of a butylated hydroxytoluene solution (1% in ethanol) and 500 μl of HPLC grade water and stored at −80°C. Ten microliters of the internal standard and five hundred microliters of alkaline hydrolysis solution (1 ml of 50% (w/w) KOH, 1 ml of water, and 10 ml methanol) were added to freshly thawed hippocampus. The resulting mixture was vortexed and incubated at 37°C for 60 min to release esterified isoprostanes. The reaction was stopped with 100 μl of formic acid 0.05% (v/v) and acidified with 90 μl of hydrochloric acid 5 N. The tubes were then extracted twice with 1.5 ml of hexane. The organic phase was discarded. The aqueous phase was then extracted three times with 1.5 ml of 3:1 ethyl acetate:hexane. The resulting organic phases of the three extractions were combined and evaporated to dryness under nitrogen and reconstituted to 100 μl in 10% (v/v) acetonitrile and 0.01% (v/v) acetic acid in water. The chromatography was carried out using a Shimadzu Prominence system (Columbia, MD). A Kinetex XB-C18 100 Å column (100 × 3.0 mm, 2.6 μm) was used preceded by a 4.0 × 2.0 mm C18 SecurityGuard cartridge, both from Phenomenex (Torrance, CA). The column oven temperature was controlled at 30°C. The injection volume was 40 μl. The separation was performed using a gradient of three solvents at a flow rate of 0.45 ml/min. Solvent A was composed of 0.01% (v/v) acetic acid in water, solvent B consisted of 0.01% (v/v) acetic acid in acetonitrile, and solvent C was 0.01% (v/v) acetic acid in methanol. First, solvent B was held at 17% for 1 min while solvent C was at 33%. The latter was followed by a linear gradient for over 8.9 min to 13.5% of B and 58.9% of C. The next step was a linear gradient over 0.5 min to 47.5% B and 47.5% C, respectively. The previous conditions were maintained for 1.6 min and solvents B and C were decreased to 17% and 33% in 0.1 min, respectively. This last condition was maintained for an additional 4.4 min to complete the 16.5 min run. The HPLC was coupled to a 3200 QTRAP® LC/MS/MS system from AB Sciex (Concord, ON, Canada) through a Turbo V ^M^ ion source using the electrospray ionization probe operated in negative mode. Curtain gas, collision gas, ion source gas 1, and ion source gas 2 were set at 37, 7, 45, and 55, respectively. The ions spray voltage was set at −4,100 V and source temperature was set at 700°C. Prostaglandin(PG)F_2α_ and 15 series F_2_-isoprostanes(isoPs) and their internal deuterated standards, PGF_2α_-d4 and 15-F_2*t*_-IsoP-d4 were monitored in the multiple-reaction monitoring mode using the transitions 353.3→193.2 *m/z* and 357.3→197.2 *m/z*, respectively. PGF_3α_ derived from EPA were monitored at transition 351.3→219.1 *m/z*. The 8-series isoPs, 8-F_2*t*_-IsoP and their internal standard, 8-F_2*t*_-IsoP-d4, were monitored using the respective transitions 353.3 →127.0 *m/z* and 357.0 →127.0 *m/z*. Finally, 5-series F_2_-IsoPs and their internal standard, 5-F_2t_-IsoP-d11, 5-*epi*-5-F_2t_-IsoP-d11, and 5(*RS*)-5-F_2c_-IsoP-d11, were analyzed using the transitions 353.0→115.0 *m/z* and 364.6→115.0 *m/z*. Quantification was performed using Analyst® 1.4.2 software (AB Sciex) (73).

### Antibodies

We used primary antibodies to detect the following: actin (1:10,000) (Applied Biological Materials, BC, Canada), amyloid precursor protein A4 clone 22C11 (1:1,500) (Millipore, ON, Canada), ApoE (mouse) (1:2,000) (Novus Biologicals, Centenial, CO), Bax (1:1,000) (Cell Signaling Technology, Beverly, MA), Bcl2 (1:1,000) (Cell Signaling, Beverly, MA), cyclin-dependent kinase-5 (Cdk5) (1:1,000) (Santa Cruz Biotechnology, Dallas, TX), cyclooxygenase-1 (COX-1) and cyclooxygenase-2 (COX-2) (1:1,000) (Cayman Chemical, Michigan), calcium-dependent cytosolic phospholipase A2 (cPLA2) (1:1,000) (Santa Cruz Biotechnology, Dallas, TX), glycogen synthase kinase-3β phosphorylated at Ser-9 (GSK-3β pS9) (1:5,000) (Cell Signaling, Beverly, MA), Ca^2+^-independent intracellular phospholipase A2 (iPLA2) (1:1,000) (Santa Cruz Biotechnology, Dallas, TX), NFkB p65 (1:2,000) (Santa Cruz Biotechnology, Dallas, TX), p21-activated kinase (PAK1/2/3) (1:1,000) (Cell Signaling, Beverly, MA), p35 (1:1,000) (Santa Cruz Biotechnology, Dallas, TX), postsynaptic density protein 95 clone K28/43 (1:5,000) (Upstate Biotechnology, Massachusetts), septin 3 (1:20,000) (Novus Biologicals, Centenial, CO), SIRT1 (1:2,000) (Upstate Biotechnology, Massachusetts), shank1 (1:1,000) (Neuromab, Davis, CA), synaptophysin (1:20,000) (Chemicon), synaptosomal-associated protein-25 (SNAP-25) clone SMI81 (1:1,000) (Covance, Berkeley, CA), tau C (human tau total) (1:10,000) (Dako, Ontario, Canada), tau protein clone tau-13 (total) (1:5,000) (Covance, Berkeley, CA), and phosphorylated tau protein (AD2) (1:20,000) (BIO-RAD, Marnes la Coquette, France), phosphorylated tau protein clone CP13 (1:1,000) (gift from late Dr Peter Davies, Albert Einstein College of Medicine), and phosphorylated PHF-tau clone AT270 (1:5,000) (Pierce/Thermo Fisher).

### Generation of cortical tissue homogenates for protein analyses

After adding eight volumes of Tris-buffered saline (TBS) containing Complete protease inhibitor cocktail (Roche, Indianapolis, IN), 10 μg/ml of pepstatin A and phosphatase inhibitors (1 mM sodium pyrophosphate and 50 mM sodium fluoride), frozen samples were briefly sonicated (3 × 10 s) and centrifuged at 100,000×*g* for 20 min at 4°C to generate TBS-soluble intracellular and extracellular fractions (soluble fraction). The TBS-soluble pellet was sonicated in the same volume of lysis buffer (150 mM NaCl, 10 mM NaH_2_PO_4_, 1% Triton X-100, 0.5% SDS and 0.5% sodium deoxycholate) containing the same protease and phosphatase inhibitor cocktail. The resulting homogenate was centrifuged at 100,000×*g* for 20 min at 4°C to produce a lysis buffer-soluble fraction (detergent-soluble fraction). The detergent-insoluble pellets were homogenized in 175 μl of 90% formic acid followed by a short sonication (1 × 10 s). The resulting suspension (detergent-insoluble fraction) was centrifuged (10,000×*g*; 4°C x 20 min) and 50 μl of the supernatant were dried out and resuspended with 5 M guanidine diluted in 0.05 M Tris-HCl to be used for ELISA. The rest of the supernatant was dried out using a SpeedVac (Thermo Savant, Waltham, MA), solubilized in 1X Laemmli’s buffer, and processed for Western immunoblotting.

### Aß ELISA

Transgenically produced human Aβ40 and Aβ42 concentrations were measured by ELISA. Soluble Aβ40 and Aβ42 were respectively measured using human β-Amyloid [1–40] ELISA kit II and [1–42] ELISA kit, High Sensitive (WAKO, Osaka, Japan). Insoluble Aβ40 and Aβ42 were respectively measured with the human Aβ40 and Aβ42 ELISA Kit (Biosource/Invitrogen/Thermo Fisher). All ELISAs were performed according to the manufacturer’s recommendations and the plates were read at 450 nm using a Synergy HT multi-detection microplate reader (Biotek, Winooski, VT).

### Western immunoblotting

For Western immunoblotting, protein concentrations were determined using bicinchoninic acid assays (Pierce, Rockford, IL). Equal amounts of protein (20 μg of total protein per lane) were added for each sample in Laemmli’s loading buffer, heated to 95°C for 5 min before loading, and subjected to SDS-PAGE. Proteins were electroblotted onto PVDF membranes (Immobilon, Millipore, MA) before washing three times in PBS and blocking in 5% nonfat dry milk and 1% BSA in PBS-0.1% Tween-20 for 1 h. Afterward, membranes were washed three times in PBS-0.1% Tween-20 for 10 min and immunoblotted with the appropriate primary and secondary antibodies followed by chemiluminescence reagents. Phosphorylated and total tau epitopes were quantified either in TBS-soluble and detergent-insoluble fractions. Immunoblots were revealed with horseradish root peroxidase–conjugated AffiniPure donkey anti-rabbit or goat anti-mouse secondary antibodies (1:60,000 each; Jackson ImmunoResearch, West Grove, PA) and detected by enhanced chemiluminescence (Lumiglo Reserve, KPL, Gaithersburg, MD) (supplemental fig. 1). Band intensities were directly quantified using a KODAK Image Station 4000 Digital Imaging System (Molecular Imaging Software version 4.0.5f7, KODAK, New Haven, CT). Before capturing the image, the software adjusts the exposure time to achieve an optimal detection and linear intensity of the bands and to avoid saturation of the signal. Quantifications were based on the net intensity of the bands, which represents the sum of the background-subtracted pixel values in the band rectangles.

### Data and statistical analyses

Statistical analyses were performed using SAS, JMP 16, and GraphPad Prism 10 software packages with the support of the biostatistical team of the Platform of Clinical and Evaluative Research of the CRCHUQ. The threshold for statistical significance was set to *P* < 0.05. Homogeneity of variance and normality were determined for all data sets using Shapiro–Wilk normality test. When normality was verified, unpaired Student’s t-tests were used to identify significant differences between two groups. Otherwise, the Welch correction or a Mann–Whitney test was performed. When more than two groups were compared, parametric one-way ANOVA followed by Tukey’s post-hoc test were performed unless variances were different, in which case a Welch-ANOVA was done instead followed by Dunnett’s post-hoc test. When both normality and variance equality were not confirmed, a nonparametric Kruskal–Wallis test followed by Dunn’s multiple comparison was performed. Relative optical density values obtained were log transformed when needed to reduce variances and provide more normally distributed data when appropriate. A ROUT test (Q = 1%) was performed on GraphPad to determined outliers. Linear regression was used to calculate the coefficient of correlation *R*^2^, *F* and the *P*-value was obtained using a generalized linear model.

## Results

### DHA and DHA + EPA diets altered brain fatty acid profiles

We first examined whether the fatty acid content of diets (shown in Table 1) led to corresponding changes in fatty acid composition of the brain. For this purpose, the fatty acid profile was determined in the frontal cortex of all animals and expressed as % of total fatty acids (Fig. 1A–F, and supplemental fig. 2). When comparing genotypes, basal levels of DHA (Fig. 1F) were lower in 3xTg-AD mice (−9%, *P* < 0.001), whereas those of ARA (Fig. 1B) were higher (+6%, *P* < 0.0001). After consumption of the DHA diet, levels of DHA remained lower in 3xTg-AD mice than NonTg mice (−6%, *P* < 0.001). Globally (Fig. 1), both DHA and DHA + EPA diets led to an increase in n-3 PUFA (Fig. 1D–F) and a decrease of n-6 PUFA (Fig. 1B and C) in the brain of both NonTg and 3xTg-AD mice compared to the control diet and hence to higher n-3/n-6 ratios of PUFA in the brain (Fig. 1H and I). Linoleic acid remained unchanged between groups (Fig. 1A), comprising about 2.0% of the total fatty acids.

Consumption of the DHA diet increased the amount of n-3 PUFA in the brain of NonTg (DHA +14%, *P* < 0.0001; n-3 DPA +280%, *P* < 0.0001) and 3xTg-AD mice (DHA +19%, *P* < 0.0001; n-3 DPA +292%, *P* < 0.0001) compared to the control diet (Fig. 1D–F). The DHA + EPA diet increased the level of DHA (+7%, *P* < 0.001) and, more strikingly, of n-3 DPA by 7.5 fold (*P* < 0.0001) in the cortex of 3xTg-AD mice, compared to the control diet (Fig. 1D and E). EPA was not detectable in the cortex of mice fed the control diet. However, after the DHA diet, EPA levels reached (*P* < 0.0001) 0.12% and 0.14% of total brain FA in NonTg and in 3xTg-AD mice, respectively (Fig. 1D). Brain EPA levels in 3xTg-AD mice further went up (*P* < 0.001) to 0.29% of total FA after consumption of the DHA + EPA diet, comparable to controls (Fig. 1E). Moreover, 3xTg-AD animals that received the DHA + EPA diet had lower concentrations of DHA (−9%, *P* < 0.001) but more than double the amount of EPA (+108%, *P* < 0.05) and n-3 DPA (2.5-fold increase, *P* < 0.0001) in the cortex compared to those fed the DHA diet. On the other hand, the DHA diet reduced two n-6 PUFA (ARA -15%, *P* < 0.001; n-6 DPA -45%, *P* < 0.001) in NonTg and (ARA -20%, *P* < 0.001; n-6 DPA -38%, *P* < 0.001) in 3xTg-AD mice, whereas the DHA + EPA diet differentially decreased ARA (−12%, *P* < 0.001) and n-6 DPA (−66%, *P* < 0.001) (Fig. 1B–C) in 3xTg-AD mice. Finally, consumption of the DHA diet resulted in a higher DHA/ARA ratio than mice fed the control diet (NonTg, *P* < 0.01; 3xTg-AD *P* < 0.0001) (Fig. 1H). Consumption of the DHA + EPA diet, which also comprises lower DHA but higher ARA than the DHA diet, resulted in a brain DHA:ARA ratio closer to that of animals fed the control diet (*P* < 0.05 vs. control diet and *P* < 0.05 vs. DHA). The n-3 DPA:n-6 DPA ratio was higher in NonTg and 3xTg-AD mice fed DHA than in control diet (∼6-fold increase, *P* < 0.01) and even higher in 3xTg-AD mice fed DHA + EPA (and containing high n-3 DPA) than in control (25-fold, *P* < 0.0001) and DHA (4-fold, *P* < 0.001) diets (Fig. 1I). Comparison of the content of PUFA in the three diets versus in the brain of 3xTg-AD mice, confirming the preponderance of DHA in the brain, with EPA appearing only after the DHA (0.13%) and DHA + EPA (0.3%) diets (Fig. 1J). Accordingly, cortical levels of ARA negatively correlated with DHA (*P* < 0.0001, Fig. 1K).

### The brain transport coefficient of [^14^C]-DHA is lower in 3xTg-AD mice

**Fig. 2:**
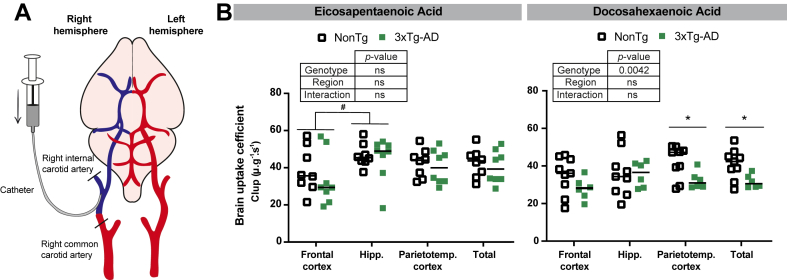
Lower brain uptake coefficient (Clup) of [^14^C]-DHA in 3xTg-AD mice. (A) schematic representation of in situ *cerebral perfusion* technique. (B) brain uptake coefficient of [^14^C]-DHA was lower in 3xTg-AD mice than in controls, whereas no such difference was observed for [^14^C]-EPA. Data are expressed in μl.g^−1^.s^−1^ and presented as means ± SEM (n = 6–9 mice per groups). Statistical comparison between NonTg and 3xTg-AD mice (14 months) was performed using unpaired Student’s *t* test, ∗*P* < 0.05, comparison between genotype in the same region; #*P* < 0.05 comparison between regions. Comparisons from two-way ANOVA are shown in boxes (factors: genotype and brain regions). Clup, brain transport coefficient or initial brain uptake clearance; DHA, docosahexaenoic acid; EPA, eicosapentaenoic acid; g, gram; Hipp., hippocampus; NonTg, nontransgenic mice; Parietotemp., parietotemporal; s, seconds; SEM, standard error mean; 3xTg-AD, transgenic mice.

To assess the brain uptake of EPA and DHA in 3xTg-AD compared to NonTg mice, we employed the in situ brain perfusion technique (Fig. 2A) (10, 74, 75, 76). First, uptake values (Clup) for both EPA and DHA were similar, as previously reported, ranging between 20 and 60 μlg^-1^s^-1^ in all animals (Fig. 2B), similar to a previous report (10). The Clup of [^14^C]-EPA was lower in the frontal cortex reached significant when compared to the hippocampus (Fig. 2B). Transport rates of [^14^C]-DHA across the BBB were slower in the parieto-temporal cortex and the total brain of 3xTg-AD mice compared to NonTg mice (effect of genotype, *P* = 0.0042) (Fig. 2B). No such difference was detected to the uptake rate of [^14^C]-EPA between 3xTg-AD and NonTg mice (Fig. 2B).

### DHA and DHA + EPA diets modulated hippocampal levels of PGF_3α_ and isoprostanes in 3xTg-AD mice

F_2_-IsoP isomers are formed from the radical-mediated peroxidation of ARA esterified in phospholipids, whereas the enzymatic conversion of EPA by the COX pathway leads to the generation of the 3-series of prostaglandins, such as PGF_3α_ (63, 77, 78). Table 2 shows the levels of isoprostanes and prostaglandins measured in the hippocampus of mice following dietary treatments. First, our study showed that n-3 PUFA supplementation led to the appearance of PGF_3α_ in the brain of both NonTg and 3xTg-AD mice. By contrast, DHA and DHA + EPA diets decreased 5-*epi*-5-F_2t_-IsoP levels in 3xTg-AD (−23%, *P* < 0.01; −18%, *P* < 0.05; respectively), but not in NonTg mice (Table 2). Thus, 5-*epi*-5-F_2t_-IsoP positively correlated with DHA levels (*F* = 7.19, *P* < 0.01) and negatively with ARA levels (*F* = 5.96, *P* = 0.018) in the brain (supplemental fig. 3). We also observed lower concentrations of 15-*epi*-PGF_2α_ and PGF_2α_ in the brain of 3xTg-AD mice fed the DHA diet comparatively to NonTg mice fed the same diet (*P* < 0.05). Finally, no significant changes for other F_2_-isoP isomers were reported.

**Table 2:** Levels of F_2_-isoprostanes and prostaglandins in the hippocampus of NonTg and 3xTg-AD mice

	NonTg		3xTg-AD		
	Control	DHA	Control	DHA	DHA + EPA
	N = 11	N = 10	N = 12	N = 14	N = 13
Isoprostanes					
5-series					
5-F_2t_-IsoP	1.3 ± 0.1	1.2 ± 0.1	1.2 ± 0.1	1.1 ± 0.0	1.2 ± 0.1
5-epi-5-F_2t_-IsoP	1.9 ± 0.1	1.8 ± 0.1	2.2 ± 0.1	1.7 ± 0.1^b^	1.8 ± 0.1^a^
5 (*RS*)-F_2c_-IsoP	8.9 ± 0.7	8.2 ± 0.7	9.2 ± 0.6	7.8 ± 0.5	8.5 ± 0.6
8-series					
8-F_2t_-IsoP	0.9 ± 0.0	0.9 ± 0.0	0.8 ± 0.0	0.9 ± 0.0	0.9 ± 0.0
15-series					
15-epi-15-F_2t_-IsoP	1.7 ± 0.1	1.7 ± 0.1	1.5 ± 0.1	1.6 ± 0.1	1.8 ± 0.3
15-F_2t_-IsoP_2t_	4.0 ± 0.3	4.5 ± 0.6	3.6 ± 0.4	3.3 ± 0.3	3.3 ± 0.4
15-epi-PGF_2α_	9.0 ± 0.5	10.1 ± 1.3	7.3 ± 0.6	6.9 ± 0.8^d^	7.1 ± 0.8
Prostaglandins					
PGF_2α_	71.0 ± 7.3	95.3 ± 17.2	58.7 ± 10.0	51.9 ± 8.5^d^	53.1 ± 9.1
PGF_3α_	nd	9.0 ± 1.3^c^	nd	7.2 ± 1.3^c^	9.8 ± 1.9^c^

iPF, isoprostanes F; nd, not detected; PGF, prostaglandin F.

Concentration (pg/mg of tissue) values are expressed as means ± S.E.M. Statistical analyses were performed using an ANOVA followed by Tukey-Kramer posthoc.

a *P* < 0.05.

b *P* < 0.01.

c *P* < 0.0001, versus control diet (same genotype).

d *P* < 0.05 versus NonTg (same diet).

### The DHA + EPA diet COX-1 and COX-2 levels but the DHA diet reduced cPLA_2_ levels in 3xTg-AD mice

**Fig. 3:**
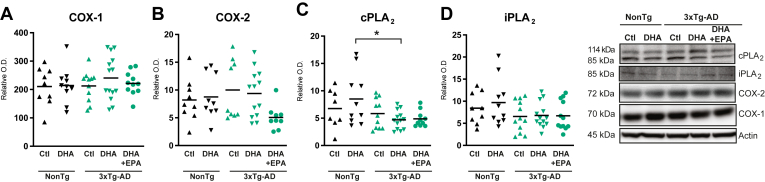
DHA + EPA diet did not significantly affect COX-1 and COX-2 levels in the parieto-temporal cortex of 3xTg-AD mice. (A) COX-1, (B) COX-2, (C) cPLA_2_, and iPLA_2_ (D) levels were not affected by DHA + EPA diet compared to control and DHA diets. However, after DHA supplementation, cortical levels of cPLA2 were reduced in 3xTg-AD mice, compared to nontransgenic mice. Immunoblot analyses in soluble fraction from the parieto-temporal cortex of nontransgenic and 3xTg-AD mice are shown in the *right* panel. Data are presented as means ± SEM; each dot represents individual values (n = 5–14 mice per group). Statistical analyses were performed using an ANOVA followed by Tukey-Kramer post-hoc tests, ∗*P* < 0.05. COX-1 and -2, cyclooxygenase 1 and 2, cPLA_2_, calcium-dependent cytosolic phospholipase A_2_; DHA, docosahexaenoic acid; EPA, eicosapentaenoic acid; iPLA_2_, calcium-independent intracellular phospholipase A_2_; SEM, standard error mean.

COX isoenzymes, COX-1 and COX-2 catalyze the formation of prostaglandins, including prostaglandin E2 (PGE_2_) and F2 (PGF_2α_) from ARA, and PGE_3_ and PGF_3α_ from EPA (79). Phospholipases A_2_ (PLA_2_) are a diverse family of hydrolytic enzymes, including calcium-dependent cPLA_2_ and calcium-independent iPLA_2_ that catalyze the cleavage of FA from the *sn-2* position of membrane glycophospholipids to generate lysophospholipids and free FA (80, 81, 82, 83). Reduced *postmortem* activity of cPLA_2_ and iPLA_2_ was reported in parietal and temporal cortices of AD subjects, as well as cPLA_2_ activity in the hippocampus (65, 84, 85). In the present study, none of the diets significantly affected COX-1 or COX-2 levels (Fig. 3A) and the only difference observed was that the levels of cPLA_2_ contrasted between 3xTg-AD and NonTg animals under the DHA diet (−45% lower in 3xTg-AD, *P* < 0.05; same diet) (Fig. 3C). No change in iPLA_2_ levels were detected in all groups (Fig. 3D).

### DHA and DHA + EPA diets decreased levels of insoluble and phosphorylated tau but did not modulate cortical Aβ levels in 3xTg-AD mice

**Fig. 4:**
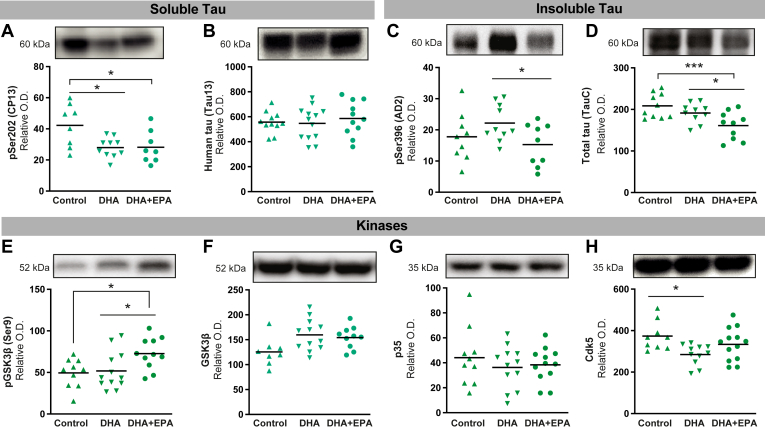
DHA + EPA diet reduced soluble and insoluble phospho-tau levels in the parieto-temporal cortex of 3xTg-AD mice. In soluble fractions, (A) phosphorylated tau (epitope Ser202) decreased following both DHA and DHA + EPA supplementations compared to control diet, but (B) levels of human total tau (epitope tau13) were unchanged. In detergent-insoluble fractions, (C) DHA + EPA supplementation decreased phosphorylated tau (epitope Ser396) compared to DHA supplementation while (D) only DHA + EPA supplementation decreased total tau (epitope tauC) levels in comparison to control diet. (E) levels of pGSK3β (phosphorylated at Ser-9, inactivated form) were upregulated with DHA + EPA supplementation while (F) total GSK3β were not modified with diets. *G*, p35 levels were not affected by diets and (H) only DHA diet decreased Cdk5 kinase. Data are presented as means ± SEM; each dot represents individual values (n = 5–14 mice per group). Statistical analyses were performed using an ANOVA followed by Tukey-Kramer post-hoc tests (∗*P* < 0.05; ∗∗∗*P* < 0.01). Cdk5, cyclin-dependent kinase 5; DHA, docosahexaenoic acid; EPA, eicosapentaenoic acid; Ser, serine; GSK3β, glycogen synthase kinase-3β.

We investigated whether DHA and EPA modulate tau pathology in 3xTg-AD animals. Both DHA and DHA + EPA significantly reduced phosphorylated tau levels measured in soluble fractions (−34%, *P* < 0.05) (Fig. 4A), without altering total tau (Fig. 4B). On the other hand, levels of insoluble phosphorylated tau were lower in animals fed DHA + EPA in comparison to the DHA group (−31%, *P* < 0.05) (Fig. 4C). Interestingly, the DHA + EPA diet led to a statistically significant downregulation in the amount of total tau protein in the insoluble fraction from the cortex of 3xTg-AD mice versus control (−23%, *P* < 0.001) and DHA (−16%, *P* < 0.05) diets (Fig. 4D). Accordingly, levels of tau (pSer202) tended to negatively correlate with DHA (*F* = 1.79, *P* = 0.194) and positively correlated with ARA (*F* = 4.86, *P* < 0.037) (supplemental Fig. 3). We next assessed whether a change in kinases could be involved in the changes in the tau phosphorylation status. While total GSK3β was not affected by diets, we observed a significant increase in the inactivated form of GSK3β (phosphorylated at Ser-9) in 3xTg-AD mice under the DHA + EPA diet compared to those who received control or DHA diets (−49%, *P* < 0.05; −40%, *P* < 0.05; respectively) (Fig. 4E–F). The DHA + EPA diet did not impact total Cdk5 levels nor p35, but mice fed the DHA diet presented lower Cdk5 levels comparatively to control diet (−24%, *P* < 0.05) (Fig. 4G–H).

We next measured Aβ in the same fractions of soluble and insoluble proteins of the parieto-temporal cortex. In accordance with previous studies (86, 87, 88), higher concentrations of Aβ were found in 3xTg-AD females, compared to males (Table 3). Thus, females and males had to be analyzed separately. As shown in Table 3, DHA and DHA + EPA diets did not significantly alter the concentrations of Aβ40, Aβ42, or Aβ42*/*40 ratios in any of the fractions tested (soluble and detergent-insoluble).

**Table 3:** Cortical Aβ levels of 3xTg-AD mice after DHA or DHA + EPA supplementation

	Control; n	DHA; n	DHA + EPA; n	Kruskal-Wallis (χ^2^)	Multivariate analyses
Soluble fraction fg/μg proteins					
Males					
Amyloid-β_42_	28.4 ± 4.4; 7	20.3 ± 7.1; 6	42.5 ± 13.0; 6	0.7961	Higher in females *P* < 0.0001
Amyloid-β_40_	23.8 ± 2.8; 7	22.2 ± 4.7; 6	23.9 ± 3.9; 6	0.4411	Higher in females *P* < 0.0001
Amyloid-β_42/40_	1.2 ± 0.2; 7	1.0 ± 0.1; 6	1.7 ± 0.4; 6	0.3577	Higher in females *P* = 0.0490
Females					
Amyloid-β_42_	606.9 ± 151.0; 3	390.0 ± 61.7; 6	632.7 ± 287.2; 4	0.2874	
Amyloid-β_40_	344.9 ± 231.6; 3	212.6 ± 80.3; 6	100.8 ± 15.5; 4	0.5101	
Amyloid-β_42/40_	2.4 ± 1.2; 3	2.5 ± 0.4; 6	6.2 ± 2.2; 4	0.4925	
Insoluble fraction fg/μg tissue					
Males					
Amyloid-β_42_	9.6 ± 2.8; 7	5.0 ± 1.0; 5	21.9 ± 7.3; 5	0.1205	Higher in females *P* < 0.0001
Amyloid-β_40_	1.6 ± 0.6; 7	0.9 ± 0.1; 5	3.6 ± 1.1; 5	0.2159	Higher in females *P* < 0.0001
Amyloid-β_42/40_	8.1 ± 1.5; 7	5.8 ± 0.4; 5	7.5 ± 1.3; 5	0.6449	
Females					
Amyloid-β_42_	125.0 ± 25.2; 3	154.1 ± 32.4; 6	138.3 ± 24.4; 4	0.1924	
Amyloid-β_40_	127.4 ± 46.1; 3	67.4 ± 14.7; 6	48.6 ± 14.8; 4	0.9033	
Amyloid-β_42/40_	1.2 ± 0.3; 3	2.6 ± 0.4; 6	3.8 ± 1.6; 4	0.0855	Higher in males *P* = 0.0003

DHA, docosahexaenoic acid; EPA, eicosapentaenoic acid.

Values are expressed as mean ± SEM.

### Favorable effects of DHA and EPA on synaptic proteins levels

Finally, we investigated the effects of dietary treatments on synaptic proteins known to be altered in AD (1, 18, 19, 89) (Table 4). We found that DHA and DHA + EPA diets led to higher SNAP25 (+28% and +25% respectively, *P* < 0.05) and septin 3 (+54% and +42% respectively, *P* < 0.01) levels in 3xTg-AD mice comparatively to mice fed the control diet (Table 4). DHA and DHA + EPA diets increased the level of PAK1/2/3 by 58% (*P* < 0.001) and 83% (*P* < 0.001), respectively, in the cortex of 3xTg-AD mice (Table 4).

**Table 4:** Levels of proteins from parieto-temporal cortex of NonTg and 3xTg-AD mice

	NonTg		3xTg-AD		
	Ctl	DHA	Ctl	DHA	DHA + EPA
Relative O.D					
Synaptic proteins					
SNAP25	333.0 ± 19.2	393.5 ± 27.1	341.0 ± 22.6	435 ± 21.4^a^	427.9 ± 24.9^a^
Septin 3	58.6 ± 5.4	85.0 ± 15.7	52.7 ± 5.1	81.1 ± 6.0^b^	74.6 ± 5.1^b^
PSD-95	19.8 ± 2.2	14.3 ± 1.5^a^	18.2 ± 1.6	16.8 ± 1.1	15.5 ± 2.0
PAK1/2/3	23.1 ± 4.0	25.2 ± 6.4	17.8 ± 2.8	28.2 ± 2.2^c^	32.6 ± 2.0^c^
Synaptophysin	35.6 ± 5.6	32.5 ± 5.5	38.5 ± 3.6	35.7 ± 3.8	34.1 ± 3.6
Shank1	45.7 ± 11.7	39.2 ± 7.8	50.1 ± 12.6	52.2 ± 4.8	37.6 ± 5.7
Apoptosis related					
Bcl-2	112.0 ± 11.9	149.2 ± 12.8	97.8 ± 6.3	114.2 ± 7.1^d^	113.0 ± 13.0
Bax	180.0 ± 20.6	165.4 ± 15.1	112.7 ± 6.5^d^	138.6 ± 10.8	148.6 ± 7.4^a^
NF-kB	67.6 ± 11.5	105.1 ± 18.4	110.4 ± 19.1	109.5 ± 11.3	82.4 ± 7.3
Others					
ApoE	12.9 ± 11.5	11.4 ± 1.6	9.7 ± 1.4	11.5 ± 1.3	13.4 ± 1.4
SIRT1	3.6 ± 0.8	2.3 ± 0.4	2.6 ± 0.4	2.6 ± 0.3	3.5 ± 0.3

3xTg-AD, triple transgenic mouse model of Alzheimer's disease; ApoE, apolipoprotein E; Bax, Bcl-2-associated X protein; Bcl-2, B-cell lymphoma 2; NF-kB, nuclear factor-kappa B; NonTg, non-transgenic; O.D., optical density; PAK1/2/3, p21 activated kinase; PSD-95, postsynaptic density 95; SIRT1, sirtuin 1 protein; SNAP25, synaptosomal-associated protein 25.

Values are expressed as means ± S.E.M. normalized to actin; N = 8–14.

a *P* < 0.05.

b *P* < 0.01.

c *P* < 0.001, versus mice with the same genotype but fed control diet.

d *P* < 0.01,versus NonTg mice on the same diet.

## Discussion

The ultimate goal of modifying nutritional risk factors is to prevent or slow neurodegeneration before the clinical diagnosis of AD is made. Among widely available nutrients, n-3 PUFA have become a focus of interest due to their capacity to accumulate in the brain and to exert potentially neuroprotective actions in the rodent models of AD or PD (6, 13, 16, 18, 19). However, a central question remains as to whether a supplement enriched mostly in EPA presents a real advantage over a supplement enriched mostly with DHA in preventing brain pathology. The aim of the present study was to answer that question using PUFA-enriched diets and a model of AD neuropathology. The present work is consistent with the main following conclusions: (i) DHA supplementation increased the concentrations of DHA in the brain, while reducing ARA and n-6 DPA. The addition of EPA on top of DHA further increased cortical EPA and n-3 DPA levels by a large margin. (ii) EPA or DHA intake led to the appearance of anti-inflammatory PGF_3α_, which was undetectable in control diet-fed animals. (iii) The brain uptake coefficient of [^14^C]-DHA was lower in 3xTg-AD than in nontransgenic mice, whereas that of [^14^C]-EPA was unchanged. (iv) Dietary DHA decreased brain soluble phospho-tau, whereas the addition of EPA also led to reduced insoluble total and phospho-tau, both key pathological markers of AD. (v) Intake of DHA or DHA-EPA increased the levels of synaptic proteins septin 3 and PAK in the cortex of 3xTg-AD mice.

### Effects of dietary treatments on brain fatty acids

No direct head-to-head comparison of EPA versus other n-3 PUFA, such as α-linolenic acid or DHA, has yet been conducted in AD models. This is because most high-EPA formulations from fish oil also contain some DHA, most DHA supplements contain EPA. Here, we utilized a custom high-EPA formulation from Ocean Nutrition Canada Ltd., under a microencapsulation process into gelatin beads that has been designed to preserve long chain PUFA for months when incorporated into food (90, 91, 92). Such microencapsulated formulations protect fatty acid molecules from oxidation to which DHA and EPA are prone due to the presence of several double bonds in their structure. As expected, the DHA-enriched formulation utilized here contained a small amount of EPA. The initial intent was to develop a diet containing high EPA and low DHA for a head-to-head comparison with a high-DHA low-EPA diet. However, determination of the fatty acid profiles in the final pelleted rodent diets revealed that the high-EPA formulation contained only slightly lower amounts of DHA (−19%) than the high-DHA diet. Therefore, the present study allowed us to study the impact of a very high dose of EPA on top of a high-DHA diet in an animal model of AD neuropathology. Although such a paradigm cannot be used to fully isolate the effects of a supplementation of EPA alone versus DHA alone, it is more representative of the real-life use of EPA supplements, which typically also contain DHA, and vice versa.

Our results show the profound effects on the brain fatty acid profile of a three-month dietary treatment varying long chain PUFA content. Supplementation with DHA increased the levels of DHA in the brain of both NonTg and 3xTg-AD mice, as previously shown repeatedly in rodents (10, 15, 16, 17, 18, 19, 93). In addition, the DHA-enriched diet increased cerebral EPA and n-3 DPA, also consistent with previous reports (10, 19, 94). Retroconversion of DHA into EPA may also have contributed (95, 96, 97, 98). An alternative hypothesis is that DHA supplementation would slow EPA metabolism, thereby leading to the accumulation of EPA in plasma and organs (96). However, brain EPA may have been provided by the small amount of EPA in the DHA diet. Indeed, the relative increase of EPA levels in the brain was proportional to EPA levels in the three diets to which the animals were exposed. Still, the present study is in agreement with previous reports indicating that dietary intake of a DHA supplement increases brain DHA but also lead to higher cerebral levels of other n-3- PUFA (10, 15, 16, 17, 18, 19, 93).

This study provides new insights into the effects of EPA-enriched supplementation, an area that has been rarely investigated in the past. Although a previous kinetic study using infused unesterified [^13^C]-EPA indicated that EPA can accumulate in the brain in the form of DHA (98), in our study paradigm, the high EPA intake translated into greatly increased levels of EPA and n-3 DPA in the brain, much higher than with the DHA diet. In addition, supplementation of EPA on top of DHA led to significantly lower DHA levels in the brain than supplementation with DHA only. In other words, with a daily oral dose of DHA remaining relatively constant, increasing the dose of EPA from 0.4 g/kg to 9.2 g/kg decreased brain DHA levels. This may be attributed to a metabolic bottleneck occurring at the stage of n-3 DPA, preventing further conversion to DHA or potentially to an as-yet unidentified competitive mechanism. It may also results from other subtle differences in DHA, ARA, and n-3 DPA in the diet formulations, as the latter two were higher in the DHA + EPA diet. A previous study showed that when β-oxidation is inhibited, the brain compensates in part by converting EPA to n-3 DPA, but not DHA (12). Another report shows that supplementation with EPA does not affect DHA levels in lipid fractions of the blood, (99). Conversely, retroconversion of n-3 DPA into EPA cannot be ruled out, as shown previously (99, 100, 101). Moreover, given the extremely low basal cerebral levels of EPA, the finding that a very high dose of dietary EPA resulted in only a two-fold increase in brain EPA supports the hypothesis that the capacity of the brain to retain EPA is limited because this n-3 PUFA is rapidly converted or utilized in the brain or peripheral organs (38, 98). These findings indicate that although there is complex interplay between the long-chain PUFAs in the diets, EPA supplementation is more effective at elevating brain EPA levels, while DHA supplementation should be used when the main aim is to increase brain DHA concentrations.

Beyond their effect on brain n-3 PUFA levels, these results shed light on differences in the ability of dietary DHA and EPA to lower n-6 PUFA concentrations and alter the n-3 DPA:n-6 DPA ratio. Previous studies have consistently documented the capacity of DHA intake to lower brain ARA levels as observed here (15, 17, 18, 19, 93). Indeed, n-3- and n-6 PUFA share enzymatic pathways and cerebral DHA and ARA compete for residency in membrane phospholipids (102), which is confirmed here by a strong inverse relationship between levels of DHA and ARA in the brain. As the dietary intervention leads to higher brain concentrations of DHA, less ARA can be incorporated into membrane phospholipids. It was previously suggested that the slower uptake of DHA across the BBB than ARA potentially also limits transfer of DHA from the brain to the blood, which would contribute to preferential retention of DHA within the brain (103, 104, 105). Dietary DHA intake was also shown to reduce the formation of the ARA-derived metabolite n-6 DPA (106). Here, despite having less impact on brain ARA, the DHA + EPA diet was more efficient in decreasing n-6 DPA and increasing n-3 DPA than the DHA diet. Interestingly, despite levels of ARA being relatively much more abundant than EPA, brain levels of n-6 DPA are less tied to its precursor ARA than to EPA, which competes with ARA for elongase-2 and -5 enzymes and leads to the preferential formation of n-3 DPA (38). Of note, the relatively high level of n-6 DPA in the brain of the animals fed the control diet likely results from the low level of omega-3 fatty acids in the control diet (18, 94). While DHA strongly competes with ARA in the brain for membrane residency, cerebral EPA predominantly competes with ARA for elongases 2 and 5, thereby increasing the n-3 DPA:n-6 DPA ratio. However, this surplus of n-3 DPA did not convert into more cerebral DHA. Overall, the present study suggests that high EPA supplementation is less prone than DHA to reduce ARA levels but is remarkably effective at elevating the n-3 DPA:n-6 DPA ratio in the brain.

### Uptake of EPA and DHA through the BBB

It has been hypothesized that lower levels of DHA and EPA in the brain can result from reduced brain uptake, thereby leading to cognitive impairment (3, 107). In situ brain perfusion data revealed that unesterified DHA and EPA readily both cross the BBB with Clup values around 40 μl .g^−1^ .s^−1^, in the same range as diazepam, a freely diffusible lipophilic drugs. These values, similar for DHA and EPA, are in accordance with previous studies (10, 108) and contradicts the possibility that a lower uptake of EPA across the BBB explains why this n-3 PUFA does not accumulate as much as DHA in the brain. Interestingly, the DHA uptake rate was lower in the brain of 3xTg-AD mice, particularly in the parieto-temporal cortex, compared to NonTg mice. Such a difference was not detected for EPA, which may reflect differences between EPA and DHA uptake mechanisms in relation to AD neuropathology (40, 41). It is possible that DHA accumulation in the brain relies more heavily on proteins such as FABP5 (fatty acid binding protein 5) and Mfsd2a (major facilitator superfamily domain-containing protein 2A) (109, 110). The reduced expression of these proteins has been associated with lower DHA levels in the brains of both AD patients and animal models (111, 112). In contrast, uptake of EPA, which is incorporated into cell membranes to a lesser extent than DHA, may rely less on these specific transporters. In sum, a slower rate of uptake of DHA in the brain can offer an explanation for the lower concentrations of DHA found in the cortex of the 3xTg-AD mice, observed here and previously (108, 113). Since the rate of brain uptake of EPA was not impaired in 3xTg-AD mice, EPA supplementation could therefore better maintain its efficacy in AD patients.

### Modulation of oxidative stress and prostaglandins by diets: Dietary n-3 PUFA increases brain PGF_3α_

Isoprostanes are produced nonenzymatically in vivo from the free radical-catalyzed peroxidation of PUFA (63). In particular, ARA-derived F_2_-isoPs and its most studied isomer, 15-F_2*t*_-IsoP, also referred as the classical 8-iso-PGF_2α_, are known reliable biomarkers of oxidative stress (63, 114, 115). In the present study, we observed no transgene-related difference in the levels of most F_2_-IsoPs, contrasting with a previous report of higher 8,12-iso-iPF_2α_-VI (5(*RS*)-5-F_2c_-IsoP) levels in the cortex and hippocampus of Tg2576 APP mice correlating with amyloid deposits (116). Thus, 3xTg-AD mice do not recapitulate the elevated levels of F_2_-IsoPs that have been measured in the brain, CSF, plasma, and urine from individuals with mild cognitive impairment or AD (63, 117, 118, 119, 120, 121, 122, 123). In contrast, we observed slightly lower 15-epi-PGF_2α_ levels in 3xTg-AD versus control mice, reaching statistical significance only in DHA-fed animals.

Given that F_2_-IsoPs are formed when ARA undergoes lipid peroxidation, they can potentially be modulated by dietary PUFA (55, 63). Dietary intake of n-3 PUFA has been reported to reduce lipid oxidation and COX-2 expression while modulating brain and blood concentrations of pro-inflammatory cytokines (18, 33, 124, 125). Previous studies have shown that n-3 PUFA supplementation can reduce F_2_-IsoPs in tumors in mice (78) and the plasma of humans (55, 77). Such an effect was only found here for 5-epi-5-F_2*t*_-IsoP in 3xTg-AD mice, which was decreased by both DHA and DHA + EPA diets. Two key enzymes for eicosanoid production were assessed by Western blots, cPLA_2_ and iPLA_2_, for which changes have been reported in post mortem AD brains (65, 85), but no differences were found here between groups. However, a more complete analysis of brain eicosanoid lipidome and enzymes involved, including sPLA_2_-IIA, would be necessary to draw a clear pattern of changes in the enzymatic machinery.

The most notable effect of n-3 PUFA here was the increased production of PGF_3α_ in the brain, a prostaglandin derived from EPA, which was undetectable in animals fed with the control diet. To our knowledge, this is the first evidence of dietary-produced PGF_3α_ in the cerebral tissue. This finding is in agreement with previous observations, where PGF_3α_ was below the detection limit in the brain of rats fed a diet without EPA or DHA (126) and found in higher concentrations in the tumors of animals fed a n-3 PUFA-enriched diet (78). Overall the general pattern of changes observed indicate that pro-inflammatory ARA-derived F_2_-isoprostanes were either decreased or unchanged, while anti-inflammatory EPA-derived PGF_3α_ was greatly increased in the brains of mice following DHA and DHA + EPA diets. In parallel, we observed lower levels of PGF_2α_, 15-epi-PGF_2α_, and cPLA_2_ in the hippocampus of 3xTg-AD mice than in non-transgenic mice following the DHA-enriched diet. Therefore, this suggests that, although PGF_2α_/15-epi-PGF_2α_ and ARA are connected by enzymatic/nonenzymatic pathways, their concentrations are not automatically interdependent. The high quantities of ARA in the brain are probably in excess and enzymes like cPLA_2_ become the limiting step in prostaglandin and isoprostane production. In contrast, PGF_3α_ levels more closely follow the brain concentrations of EPA, supporting that, in this pathway where the PUFA precursor is rare, EPA is the limiting step. These results suggest that the AD transgenes impact the regulation of mechanisms linking long-chain PUFA with downstream F_2_-isoprostanes and prostaglandins. Overall, the changes in isoprostanes and PGF_3α_ concentrations reported here could underlie a previously unknown anti-inflammatory mechanism for n-3 PUFA in the brain.

### Effect of EPA and DHA on Aβ and tau pathologies in the brain

Amyloid plaque deposits and tau NFT are major neuropathological markers of AD (1, 89). DHA and DHA + EPA diets had no significant impact on Aβ levels in the parieto-temporal cortex of 3xTg-AD mice. This contrasts with previous studies which had suggested a positive effect of DHA on reducing Aβ accumulation in the brains of mouse models of AD while improving cognition and preventing dysfunction of entorhinal cortex neurons (13, 14, 17, 93). This difference could be explained by several factors: the origin, the quantity of DHA present in the diets, or the duration of the treatment. The higher levels of Aβ in females than in males, consistent with previous studies (86, 87, 88), have added a confounding variable that may have hindered the detection of diet-induced changes, in part by reducing the number of animals in each group.

The hyperphosphorylation of tau and its extensive conversion into an insoluble form are a major feature of AD pathogenesis, which correlates well with cognitive deficit (1, 2, 89). Clinical studies have reported improved cognitive outcome following n-3 PUFA administration, particularly in individuals with a mild cognitive impairment, but they provide no clear evidence of the superiority of EPA versus DHA (4, 49, 50) and of any impact on tau pathology. Preclinical studies report that DHA supplementation or endogenous conversion of n-6 PUFA into n-3 PUFA in 3xTg-AD mice reduce the levels of phosphorylated tau (17, 93), but none investigated EPA specifically. Here, both DHA and DHA + EPA diets decreased soluble phosphorylated tau levels whereas only the DHA + EPA supplementation decreased the levels of insoluble phosphorylated and total tau in the brain of 3xTg-AD mice. The phosphorylation of tau on serines and threonines is carried out by several kinases (127, 128), such as Cdk5 and GSK3β, and may account for the hyperphosphorylation of tau observed in AD. Preclinical studies suggest that inhibiting GSK3β leads to a reduction of tau phosphorylation, an improvement of memory and a decrease of axonal degeneration in transgenic mice (129, 130). The changes in the brain levels of GSK3β and Cdk5 observed here following DHA + EPA and DHA diets, respectively, may thus explain the effect of these n-3 PUFA on tau phosphorylation status. The effect of the DHA + EPA diet increasing the inactivated form of GSK3β (pSer9) was particularly notable. Given the importance of tau pathology in AD, as a diagnostic biomarker and a key pathogenic player, this effect of n-3 PUFA, and particularly of EPA on insoluble tau, may provide evidence of a disease-modifying action of these widely available nutraceuticals.

### Effect of EPA and DHA on synaptic proteins

Synaptic loss is as important as Aβ and tau pathologies in AD. Post mortem evidence indicate that synaptic loss occurs very early in the development of AD (18, 89, 131, 132). Among the proteins investigated, SNAP25, septin3, and PAK_1/2/3_ have been shown to be lower in the brain of AD patients, correlating with symptoms (89, 133, 134). In particular, the p21-activated kinase family is involved in dendritic spine defects and cognitive deficits (135), particularly PAK3 (133, 135, 136). Here, these three synaptic proteins were increased in groups of 3xTg-AD mice after intake of DHA or DHA + EPA. A protective effect of n-3 PUFA on synapse has been proposed before, although not always replicated in preclinical studies and without specifically comparing EPA versus DHA (16, 18, 20, 93, 137, 138). In AD, postsynaptic proteins such as postsynaptic density protein 95, drebrin, or shank are also downregulated in the brain (18, 139, 140, 141) but the diets used here did not affect their levels in 3xTg-AD mice. The results concerning synaptic proteins in the present study support the hypothesis of a protective effect of n-3 PUFA on synapses but do not show any additional benefit of EPA compared to DHA.

There are few limitations in this study that should be noted. Firstly, we were unable to assess the cognitive performance of the animals due to logistical reasons, focusing instead on the molecular impact of DHA and EPA. While previous reports have shown the cognitive enhancing properties of DHA (9, 14, 18, 93), it would have been relevant to investigate the effects of the chronic intake of the EPA + DHA formulation in the 3xTg-AD model at this age. Secondly, the formulation of a microencapsulated EPA, essential to its preservation in the pelleted diet, was specifically manufactured in small quantities for this study and was not standardized. The concentrations of fatty acids in the diet were determined before feeding the animals and the final doses of each long-chain PUFA are not entirely in line with what was predicted based on the starting ingredients, which complicates the interpretation of the results. However, determining the exact concentrations of brain PUFAs mitigated the impact of this variability. Lastly, the lack of significant differences in synaptic protein levels between 3xTg-AD and NonTg mice is different from what is observed in AD (89). However, it is in general agreement with previous reports in this model, except for p21-activated kinase (93, 133). This may be explained by the relatively limited Aβ pathology observed in the 3xTg-AD mouse at this age.

## Conclusion

Our findings contribute to the growing body of evidence supporting the potential benefits of n-3 PUFA supplementation in preventing AD. Previous research consistently demonstrates that dietary DHA can enhance brain DHA and reduce ARA levels. This study builds on that knowledge by showing that including a higher EPA content in the diet not only further raises brain EPA levels but also dramatically increases the n-3 DPA:n-6 DPA ratio. However, amounts of EPA in the brain remained relatively low compared to DHA and total PUFA. Furthermore, we detected PGF_3α_ in the brain only following n-3 PUFA treatment, possibly reflecting a novel anti-inflammatory mechanism of action. Although 3xTg-AD mice exhibited lower DHA uptake and lower DHA concentrations in the brain, dietary intake of n-3 PUFAs, particularly EPA, were effective in reducing insoluble tau, potentially by modulating GSK3β activity. This points to a possible disease-modifying effect that might be crucial in preventing AD. Overall, this study suggests that EPA supplementation rapidly triggers molecular mechanisms that synergize with the strong impact that DHA intake has on the balance of structural PUFA of the brain. Our results support further clinical trials to explore the efficacy of n-3 PUFA supplementation, incorporating a high dose of EPA, as a preventive strategy against AD.

## Data availability

The datasets generated and analyzed during the current study are available from corresponding author on reasonable request.

## Supplemental data

This article contains supplemental data.

## Conflict of Interest

The authors declare that they have no conflicts of interest with the contents of this article.
