# Supplementary material for: Differential impact of eicosapentaenoic acid and docosahexaenoic acid in an animal model of Alzheimer’s disease
Source: J Lipid Res. 2024 Oct 28;65(12):100682. doi: 10.1016/j.jlr.2024.100682 (PMC11650307; doi:10.1016/j.jlr.2024.100682)
Supplement: Supplemental Information [file mmc1.docx]

**SUPPLEMENTAL INFORMATION :**

**Differential impact of eicosapentaenoic acid and docosahexaenoic acid in an animal model of Alzheimer’s disease**

Méryl-Farelle Oye Mintsa Mi-mba^1,2^*, Meryem Lebbadi^1,2^*, Waël Alata^1,2^, Carl Julien^1,2^, Vincent Emond^2^, Cyntia Tremblay^2^, Samuel Fortin^3^, Colin J. Barrow^5^, Jean-François Bilodeau^2,4^, and Frédéric Calon^1,2^

^1^Faculty of Pharmacy, Laval University, Quebec (QC), Canada; ^2^Centre Hospitalier de l'Université Laval (CHUL) Research Center, Quebec (QC), Canada; ^3^Centre de recherche sur les biotechnologies marines, Rimouski (Qc), Canada ; ^4^Department of medicine, Faculty of Medecine, Laval University, Quebec (QC), Canada ;  ^5^Centre for Sustainable Bioproducts, Deakin University Geelong, Victoria, Australia

*These authors contributed equally to this work

**Supplemental Figure 1. Images of immunoblots obtained using Western immunoblotting analyses for all proteins mentioned.**

**Supplemental Figure 2. Cortical levels of other fatty acids.** Impact of DHA and DHA+EPA diets on (**A**) SFA, (**B**) MUFA, (**C**) total PUFA, (**D**) n-3 PUFA, (**E**) n-6 PUFA, and (**F**) n-3 PUFA:n-6 PUFA ratio. Data are presented as means ± SEM of relative concentrations in the frontal cortex, each dot representing an individual value (n= 8-14 mice per group). Statistical analyses were performed using an ANOVA followed by Tukey-Kramer post-hoc tests *****p*<0.0001 compared to animals with the same genotype but fed control diet. ^@^*p*<0.05; compared to animals with the same genotype but fed DHA diet, ^&^*p*<0.05; ^&&^*p*<0.01 compared to non-transgenic mice on the same diet. Abbreviations: MUFA, monounsaturated fatty acid; NonTg, non-transgenic mice; PUFA, polyunsaturated fatty acid; SEM, standard error mean; SFA, saturated fatty acid; 3xTg-AD, transgenic mice.

**Supplemental Figure 3. Opposite associations for cortical levels of DHA versus ARA, with concentrations of 5-*epi*-5-F_2t_-IsoP in the hippocampus and levels of phosphorylated tau in the parietal cortex.** Lower DHA and higher ARA content in the cortex were associated with higher levels of (**A,C**) 5-*epi*-5-F_2t_-IsoP in the hippocampus of all mice and (**B,D**) phosphorylated tau (epitope Ser202) in the cortex of 3xTg-AD mice. Abbreviations: ARA, arachidonic acid; DHA, docosahexaenoic acid; NonTg, non-transgenic mice; 3xTg-AD, transgenic mice
